# Compression Behaviors and Mechanical Properties of Modified Face-Centered Cubic Lattice Structures under Quasi-Static and High-Speed Loading

**DOI:** 10.3390/ma15051949

**Published:** 2022-03-06

**Authors:** Peng Wang, Fan Yang, Jinfeng Zhao

**Affiliations:** School of Aerospace Engineering and Applied Mechanics, Tongji University, Shanghai 200092, China; wangpeng0919@tongji.edu.cn (P.W.); jinfeng.zhao@tongji.edu.cn (J.Z.)

**Keywords:** lattice structure, load-bearing, energy absorption, plateau stress, deformation mode

## Abstract

Our previous work reported a novel lattice structure composed of modified face-centered cubic (modified FCC) cells with crossing rods introduced at the center of each cell. In this work, the proposed modified FCC lattice is further investigated to ascertain its compression behaviors under different loading rates. For this purpose, numerical simulations were carried out for compressing the two-dimensional and three-dimensional modified FCC lattice structures with different loading rates, and to compare their deformation modes and energy absorption capacity under different loading rates. In addition, lattice specimens were fabricated using selective laser melting technology and quasi-static compression experiments were performed to validate the finite element simulations. The results indicate that the proposed modified FCC lattices exhibit better load-bearing capacity and energy absorption than the traditional FCC lattices under different loading rates. Under high-speed loading, the modified FCC structure is less susceptible to buckling, and the length ratio of the central cross-rod corresponding to maximum energy absorption capacity is larger.

## 1. Introduction

The lattice structures have promising potential for applications in aerospace, automotive, and navigation due to their high specific strength [1,2], large specific stiffness [3,4], superior impact resistance [5,6], and excellent energy absorption capacity [7,8]. Existing studies [9,10,11,12] mostly focused on the relationship between structural geometries and mechanical properties. Both the quasi-static and dynamic mechanical behaviors have been extensively investigated for the lattice structures, including honeycombs [13,14], face-centered cubes (FCC) [15,16], and body-centered cubes (BCC) [17]. Although being lightweight, the porous characteristics of the lattice structures usually compromise their mechanical properties compared with their solid counterparts. In the past decade, the rapid progress of additive manufacturing technologies has made it possible to precisely fabricate the lattice structures with complex topologies, opening the door to the design and fabrication of lattice structures with ultra-lightweight and ultra-strong mechanical properties.

A considerable number of lattice designs have been proposed and proved to possess greater potential for energy absorption applications than the conventional simple cubic (SC), FCC and BCC structures. Cao et al. [18] designed a modified rhombic dodecahedron stainless steel lattice and the compression behaviors under quasi-static loading were investigated by experimental and simulation methods. Compared with conventional lattices, the proposed lattice has better mechanical properties. Guo et al. [19] designed and fabricated a composite mechanical metamaterial consisting of dual-phase architected truss lattices. The specific energy absorption, specific strength and specific stiffness of the dual-phase lattice material were investigated. Zhang et al. [20] proposed a novel re-entrant anti-tri-chiral lattice to improve both the elastic modulus and the energy absorption capacity.

As a typical lightweight structure, the dynamic compression behaviors and energy absorption properties are extremely important in their applications. Previous studies of the mechanical response of lattice structures were mainly conducted via quasi-static compressive tests [21,22]. A few dynamic studies [23,24,25] were carried out recently. Zheng et al. [26] studied the effects of cell irregularity and impact velocity on the plateau force and the deformation modes for the 2D lattices. Ruan et al. [27] obtained an empirical formula of the plateau force for the lattice structure at high-speed loading as a function of wall thickness and the impact speed. Hu et al. [28] investigated the deformation modes of the hexagonal honeycomb structure subjected to the impact loads along the two transversal directions, taking into account the effects of the relative density, the impact velocity and the cell angle. From the above review, it is found that a large part of existing research was focused on the 2D lattice structures instead of the 3D lattice structures, which may be more important for energy absorption application. In addition, the dynamic behaviors of lattice lattices were not as thoroughly investigated as their quasi-static properties. Thus, an in-depth investigation is deserved for the static and dynamic behaviors of the 3D lattice structures.

In our previous work, we proposed an innovative lattice called modified FCC lattice, which was derived from the conventional FCC lattice by inserting crossing struts at the center of the unit cell. We showed that the modified FCC lattice possesses enhanced energy absorption capacity under quasi-static compression. In this work, the dynamic compression response for the proposed modified FCC lattice is further investigated to ascertain the influence of the loading rate on its mechanical behaviors. Numerical simulations are performed using commercial software ABAQUS, to compare the deformation modes and energy absorption performance of the two-dimensional (2D) and three-dimensional (3D) modified FCC lattices with different geometric parameters under quasi-static and dynamic impact loading conditions. The organization of this article is summarized as follows. After this introduction, the design strategies and geometric configurations for the 2D and 3D models are shown in Section 2. The results of quasi-static and dynamic simulations, including the deformation modes, the load-bearing and energy-absorbing capacity, and the plateau stress, are provided in Section 3. Finally, some conclusions are summarized in Section 4.

## 2. Materials and Methods

### 2.1. Design Strategies and Geometric Configurations

**Figure 1:**
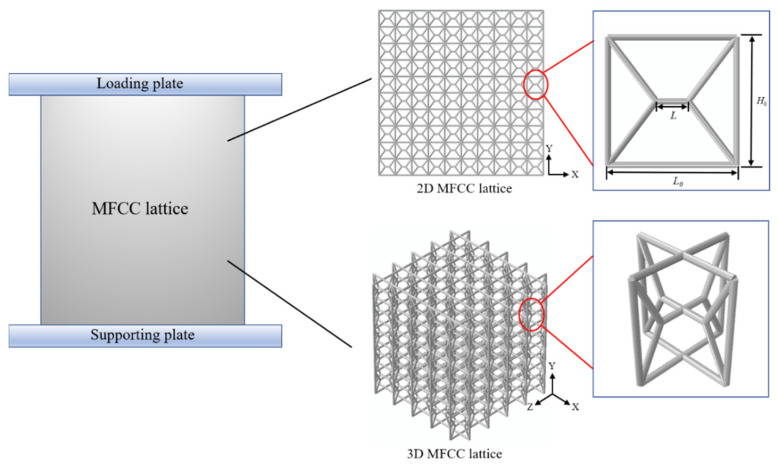
Design strategies of 2D and 3D modified FCC lattices with the enlarged view showing a unit cell.

Figure 1 shows the geometric configurations of the 2D and 3D modified FCC lattices. The modified FCC lattice is derived from the conventional FCC lattice by inserting crossing struts at the center of the unit cell. Two mutually orthogonal 2D unit cells form a 3D unit cell. The computationally efficient beam elements are used to discretize the structure. The lattice is placed between the top and bottom rigid platens. The 2D lattices are formed by 100-unit cells in a 10 × 10 array. To reduce computational costs, the 3D lattices are formed by 125-unit cells in a 5 × 5 × 5 array. The length and width of the cell and the diameter of the struts are *H*_0_ = *L*_0_ = 40 mm, *R* = 3.2 mm, respectively. The length of the cross-strut located at the center of the unit cell is L. To define the length coefficients *m* = *L/L*_0_, a series of modified FCC lattices with different m are generated by changing the length L. Therefore, the geometry configurations of the modified FCC lattices can be determined by the independent parameter m. When *m* = 0, the modified FCC lattice becomes the conventional FCC lattice, and when *m* = 1, the modified FCC lattice becomes the conventional simple cubic lattice. Therefore, the modified FCC lattice is considered to be transitional from a simple cubic lattice to an FCC lattice.

### 2.2. Material Tests and Simulation Parameters

**Figure 2:**
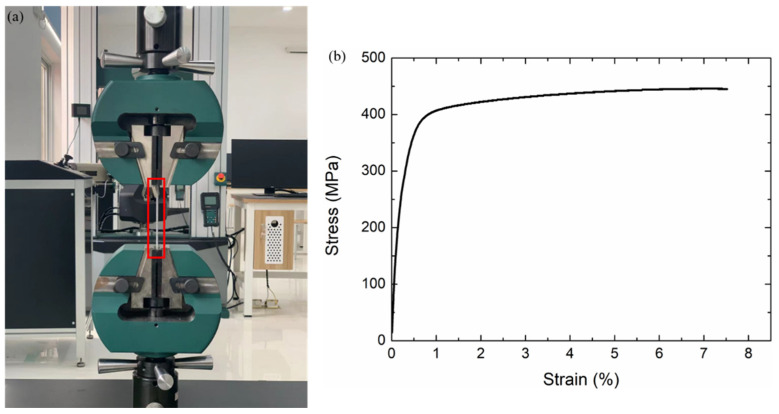
(**a**) Uniaxial tensile testing of dog-bone specimens (**b**) nominal stress versus nominal strain curve [29].

The constitutive material of the lattice structures for the numerical simulations was chosen as 316L steel (SimpNeed company, Hangzhou, China). Similar to our previous study [29], the constitutive material parameters of 316L stainless steel material were obtained by uniaxial tensile testing (Tinius Olsen 100ST, Tongji University, Shanghai, China) of dog-bone specimens, as shown in Figure 2, with Young’s modulus *E* = 210 GPa, yield stress *σ_ys_* = 279.8 Mpa, other parameters are density *ρ* = 7.98 g/cm^3^ and Poisson’s ratio is *ν* = 0.3.

For the mesh of the structure, a 2-node linear beam element B31 was adopted. Before the parametric simulation, a mesh convergence analysis was performed to ensure the accuracy and efficiency of the calculations, and the approximate size of 4 mm for the beam element was selected. In the simulations, the lattice specimen is placed between the support platen and the loading platen, where all degrees of freedom of the supporting platen are fixed. The loading platen is compressed downward at a speed of 2 m/s, with the other degrees of freedom fixed. To ensure that the 2D lattice structure is deformed only in the XY plane, the translational degrees of freedom along the Z direction are constrained for all 2D lattice specimens. The all-inclusive general contact algorithm with a friction coefficient of 0.2 is used in the model. The compression stress–strain plots are for the true stress and true (logarithmic) strain. The relations between the true strain–stress and the nominal strain–stress are given in Equations (1) and (2), where *σ_t_* and *σ_n_* are true stress and nominal stress, respectively, and *ε_t_* and *ε_n_* are true strain and nominal strain, respectively.

(1) $$\sigma_{t}=\sigma_{n}e^{\varepsilon_{t}}$$

(2) $$\varepsilon_{t}=\ln\left(1+\varepsilon_{n}\right)$$

The nominal strain is calculated from the compression displacement of the loading platen S and the height of the specimen *H*_0_ as *ε* = *S*/*H*_0_. For 2D and 3D lattices, the nominal stresses are calculated as *σ*_n_ = *F*/(*L*_0_*D*) and *σ*_n_ = *F*/$L_{0}^{2}$, respectively, where *F* and *D* are the reaction force for the loading platen and the cross-sectional diameter of the circular struts.

### 2.3. Quasi-Static Compression Experiments

**Figure 3:**
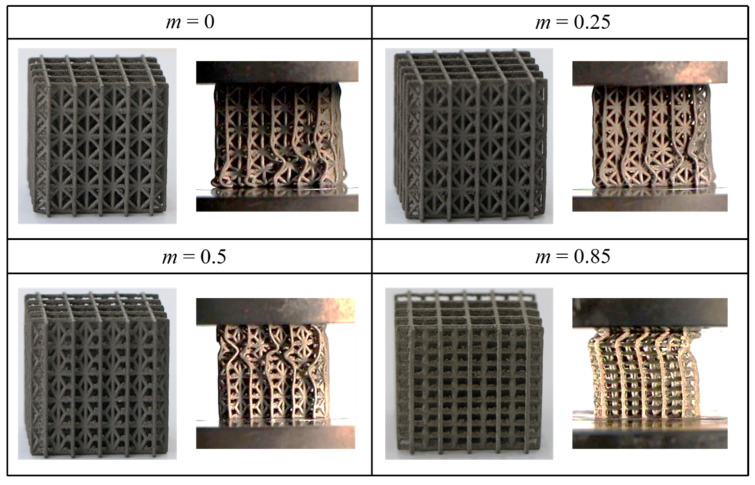
Modified FCC lattice specimens printed by SLM technology and their deformation snapshots at strain 0.1.

Quasi-static compression experiments were carried out to investigate the influence of m on the compression behaviors of modified FCC lattices. Four modified FCC lattices of different *m* were fabricated using the SLM technique (SimpNeed company, Hangzhou, China). The average particle size used for the SLM process is 30 μm. The laser power is 200 W, the thickness of the powder layer is 30 μm and the scanning speed is set as 2000 mm/s. The size of spherical powder ranges from 15 to 45 μm. All specimens were printed in the same direction that aligns with the loading direction to eliminate the deviation of material properties due to printing direction. Before the experiment, the lattice specimens were annealed in the nitrogen environment at 490 °C for 6 h to avoid the influence of residual stresses induced in the printing process. The lattice specimens were placed between the supporting platen and the loading platen, which was moved down slowly at a compression velocity of 2 mm/min. The displacement and reaction force were recorded by the sensors mounted on the loading platen to obtain stress–strain curves. The deformation modes of the lattice samples were captured by a camera (Canon 750D), as shown in Figure 3.

**Figure 4:**
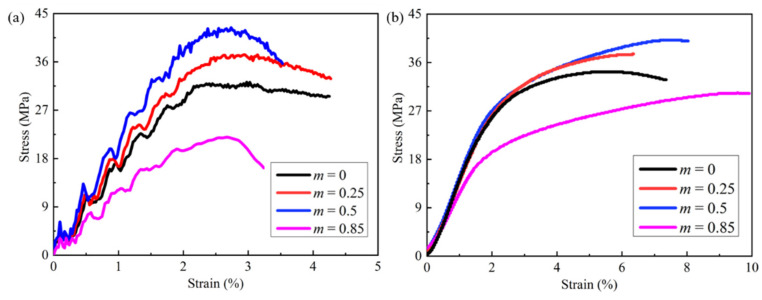
The stress–strain curves from (**a**) numerical simulations and (**b**) experimental tests for four modified FCC lattice specimens.

In our previous work, it was shown that the specific energy absorption of modified FCC lattices is larger than those of the traditional FCC and SC lattice under the quasi-static compression. In the current study, we show that the load-bearing capacity of modified FCC lattices is also higher. In Figure 3, for *m* = 0, i.e., conventional FCC lattice, the structure expands laterally during compression and presents the positive Poisson’s ratio (PPR). As the length coefficient m increases, the Poisson’s ratio of the lattice gradually decreases. When *m* = 0.25, the PPR changes to zero Poisson’s ratio (ZPR). From the stress–strain curve in Figure 4, it can be seen the results of the experimental and finite element simulations agree qualitatively, which can verify the accuracy of the numerical model. It also can be observed that when *m* ≤ 0.5, the peak stress representing the load-bearing capacity increase with m. The weakening of the PPR effect causes the shrink of the space between the struts, leading to the enhancement of the interaction between the struts and hence the elevated stress. When the m is large, e.g., *m* = 0.85, the modified FCC lattice deforms in an unstable buckling mode, leading to structural instability and a sharp drop of the peak stress. It can be noted that the experimental stress–strain curve is very smooth, different from the simulation curve, which exhibits evident oscillations. This phenomenon of stress oscillations in the FE simulations has also been reported in the previous literature [30] and is probably attributed to the idealization of the finite element models which do not consider the defects that are usually introduced during the processing of specimens in the real experiments. The defects act as triggers for the local deformation, and thus, can smooth the undulating stress–strain curves. Therefore, the oscillation of the stress–strain curve from the finite element simulations is usually larger than that from the experiments.

Usually, improving the energy absorption performance of lightweight structures is usually at the expense of their load-bearing capacity. However, the modified FCC lattice exhibits enhancements in both the energy absorption and the load-bearing capacity compared with the conventional lattices. This provides a feasible way for the design of a multifunctional energy absorber.

## 3. Results and Discussions

In order to investigate the dynamic compression behaviors and mechanical properties of modified FCC lattice structures, the explicit dynamic numerical simulations of 2D and 3D modified FCC lattice structures with different length coefficients *m* under different loading rates were carried out. The compression behaviors of the modified FCC lattice under high impact loading were compared with our previous results [29] under quasi-static loading.

### 3.1. 2D Modified FCC Lattices

**Figure 5:**
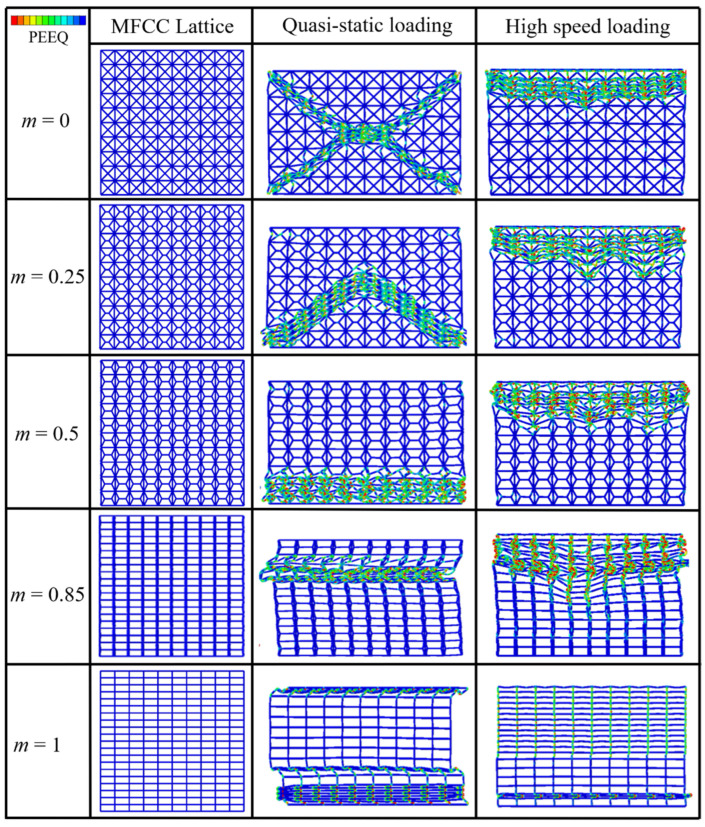
Comparison of deformation modes for 2D modified FCC lattice structures with different *m* under quasi-static [29] and high-speed of *V* = 60 m/s loading conditions.

**Figure 6:**
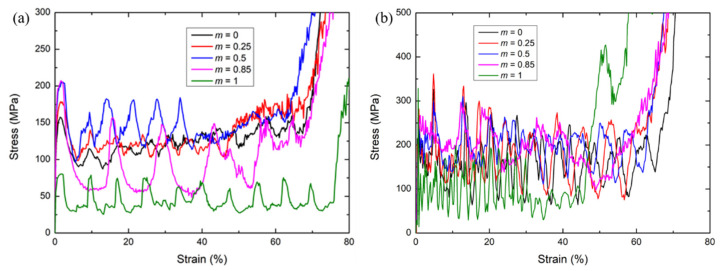
Comparison of stress–strain curves for 2D modified FCC lattice structures with different *m* under (**a**) quasi-static [29] and (**b**) high-speed loading at *V* = 60 m/s loading conditions.

The deformation modes of 2D modified FCC lattices with different m under the quasi-static compression [29] and the high-speed loading at velocity of *V* = 60 m/s, were compared in Figure 5. Under quasi-static loading conditions, it can be observed that the deformation mode of modified FCC lattices is particularly influenced by the length coefficient *m*. When m is small, e.g., *m* = 0 or 0.25, the modified FCC lattices exhibit obvious oblique deformation bands (X-shaped or V-shaped), with the deformation of struts highly centralized in the shear band. The shear bands gradually extend to neighboring layers until the densification of the full specimen. When m increases to 0.5, the shear bands gradually change to be horizontal, leading to layer-by-layer crushing deformation, which causes periodic peaks and valleys in the stress–strain curve, resulting in evident undulations of the curve (see Figure 6). When *m* is large, e.g., *m* = 0.85 or *m* = 1, the lattice specimens exhibit a deformation characteristic of global buckling. The buckling deformation mode causes the structure to collapse unstably in one lateral direction, with the rapid decrease in the stress, which is not conducive to energy absorption.

Different from the quasi-static compression, under the high-velocity loading, the modified FCC lattice does not exhibit any oblique shear bands during the entire crushing process. The deformation modes are concentrated in the horizontal shear bands perpendicular to the loading direction, similar to the form of layer-by-layer propagation of shock waves from the loading end to the support end. Especially, for *m* = 1, i.e., the simple cubic lattice case, the high loading speed diminishes the possibility of buckling deformation. These phenomena could be possibly explained by the wave theory. Under the high-velocity impact, the plastic wave speed may be lower than the loading velocity, leading to the localization of deformation near the loading platen.

The stress–strain curves of the 2D lattices obtained from the quasi-static and the high-speed loading simulations are shown in Figure 6. Compared with the quasi-static cases, the stress oscillation becomes much more evident under high-speed loading. For the high-speed loading condition, the two extreme cases of *m* = 0 and *m* = 1, i.e., the conventional FCC and SC lattices, exhibit intensive oscillations in their stress–strain curves. On the contrary, the modified FCC lattice with *m* = 0.85 shows a significantly smoother stress response, indicating a more favorable energy absorption property. It can be also observed in Figure 6b that the plateau stress of the modified FCC lattice takes the trend of first increase and then decrease as the m increases, with the maximum plateau stress at *m* = 0.85, which is larger than the value *m* = 0.5 corresponding to the maximum value for the quasi-static case. This difference is because the stress wave effect effectively avoids the buckling deformation mode. Figure 6 also shows that the initial peak stress is larger for the modified FCC lattice than the conventional FCC or SC lattices under both the quasi-static compression or high-velocity impact loading, which indicates the enhancement of the load-bearing capacity for the proposed modified FCC structure.

### 3.2. 3D Modified FCC Lattices

**Figure 7:**
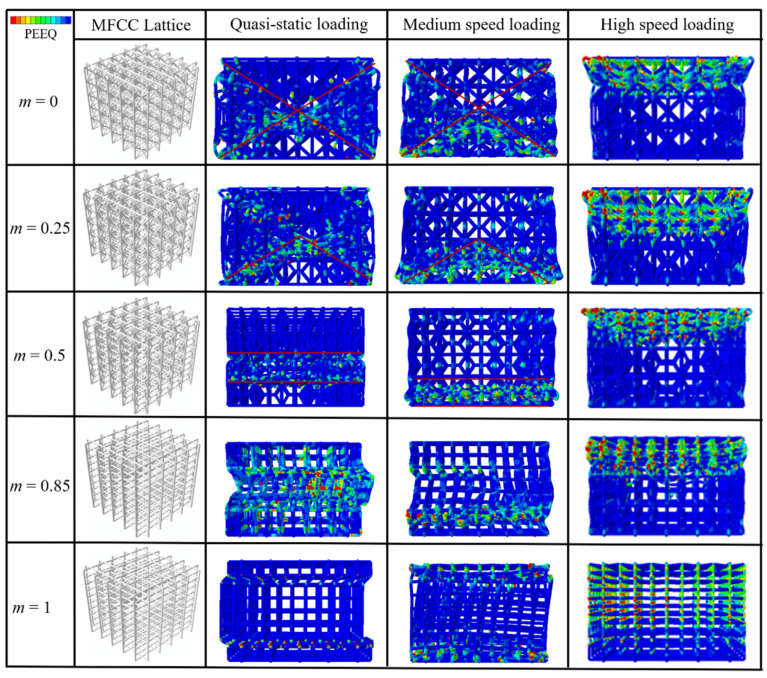
Comparison of deformation modes for 3D modified FCC lattice structures with different *m* under quasi-static [29], medium-speed of *V* = 30 m/s and high-speed of *V* = 60 m/s loading conditions.

**Figure 8:**
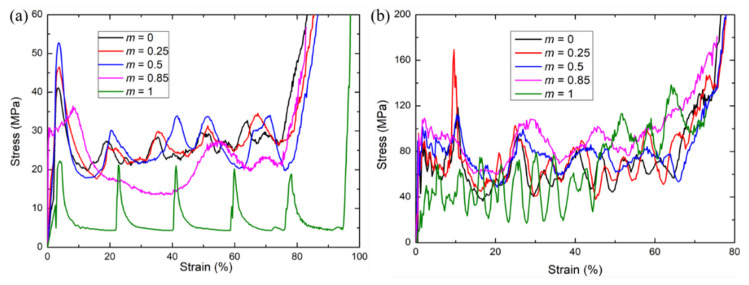
Comparison of stress–strain curves for 3D modified FCC lattice structures with different m under (**a**) quasi-static [29] and (**b**) high-speed loading at *V* = 60 m/s loading conditions.

Next, we switch our attention to the 3D modified FCC lattice structures to check if the conclusions obtained from the 2D modified FCC lattices can be applied to the 3D cases. The deformation modes and the stress–strain curves of 3D modified FCC lattice structures with different m under both quasi-static and dynamic loading at the velocity of *V* = 60 m/s are shown in Figure 7 and Figure 8, respectively. The stress–strain curves also exhibit typical stretch-dominated characteristics and can be classified into an elastic regime, plateau regime and densification regime. For the high-speed loading condition, the results are qualitatively consistent with those of the 2D modified FCC lattices, both showing that the modified FCC lattice with the length coefficient *m* = 0.85 possesses the highest energy absorption capacity with significantly larger plateau stress and comparable peak stress compared to the other structures. The deformation modes of MFCC lattice structures with different length coefficients under the medium-speed loading conditions are shown in Figure 7, which can be regarded as a transition from quasi-static to high-speed loading conditions. It can be seen that under medium-speed loading, the deformation modes of the lattice structures without global buckling deformation (with a small length coefficient) are similar to those under quasi-static conditions. However, the global buckling deformation observed for the larger length coefficient under the quasi-static loading is significantly diminished under the medium-speed loading, indicating that the loading rate significantly affects the deformation mode. The global buckling deformation mode of the simple cubic lattice and MFCC lattice with a larger length coefficient can be effectively restrained by the high-speed loading condition.

For the quasi-static compression, the 3D modified FCC lattice with *m* = 0.5 corresponds to the maximum plateau stress, which is also in agreement with the results of the 2D lattices. It is worth noting that the 3D modified FCC lattice with *m* = 0.5 possess the largest peak stress, i.e., the highest load-bearing capacity, this result is different from the 2D lattices (*m* = 0.85 corresponding to the highest load-bearing capacity). This is because the 3D modified FCC lattice with large m is vulnerable to buckling deformation under quasi-static loading conditions, leading to severe instability and a significant decrease of the stress response. However, the buckling behavior is inhibited under high-speed loading conditions, thus the lattice exhibits the same trend as the 2D lattice, i.e., the larger the length coefficient, the higher the load-bearing capacity. In addition, it also shows that the 3D lattices have less stress oscillation, showing more beneficial energy absorption capacity, since the peaks in the stress response are detrimental to the protected occupants.

### 3.3. Dynamic Plateau Stress Prediction Model

A simplified theoretical model is proposed by Reid et al. [31] to characterize the dynamic compression properties of honeycomb material based on the rigid perfectly-plastic locking (R-PP-L) material law. In their model, the predicted dynamic plateau stress is related to the impact speed *V* and the effective density $\rho_{0}$, as in the following empirical formula Equation (3):

(3) $$\sigma_{p}=\sigma_{p}^{0}+\frac{\rho_{0}V^{2}}{\varepsilon_{D}}\;$$

where $\sigma_{p}^{0}\;$ is defined as the plateau stress under quasi-static loading. Gibson and Ashby [32] expressed $\;\sigma_{p}^{0}$ and $\varepsilon_{D}\;$ as functions of the relative density $\sigma_{R}$ and the yield stress $\sigma_{y}$ as below (Equations (4) and (5)):

(4) $$\sigma_{P}^{0}=C\sigma_{y}\rho_{R}{}^{2}\;$$

(5) $$\varepsilon_{D}=1-\gamma \rho_{R}\;$$

where, $\rho_{R}=\frac{\rho_{0}}{\rho_{s}}$, $\rho_{s}$ is the base material density, *C* and *γ* are dimensionless constants.

Thus, Equation (3) can be transformed to Equation (6):

(6) $$\sigma_{P}=C\sigma_{y}\rho_{R}{}^{2}+\frac{\rho_{0}V^{2}}{\varepsilon_{D}}=C\sigma_{y}\rho_{R}{}^{2}+AV^{2}\;$$

Fitting the data from the numerical simulation, the empirical expressions of dynamic plateau stress for the five investigated lattices, i.e., *m* = 0, 0.25, 0.5, 0.85, and 1 can be obtained (Equations (7)–(11)):

(7) $$\sigma_{P_{1}}=0.45\sigma_{ys}{\rho_{R_{1}}}^{2}+0.204V^{2}$$

(8) $$\sigma_{P_{2}}=0.53\sigma_{ys}\rho_{R_{2}}{}^{2}+0.229V^{2}\;$$

(9) $$\sigma_{P_{3}}=0.62\sigma_{ys}{\rho_{R_{3}}}^{2}+0.228V^{2}\;$$

(10) $$\sigma_{P_{4}}=0.70\sigma_{ys}\rho_{R_{4}}{}^{2}+0.326V^{2}\;$$

(11) $$\sigma_{P_{5}}=0.64\sigma_{ys}\rho_{R_{5}}{}^{2}+0.182V^{2}\;$$

**Figure 9:**
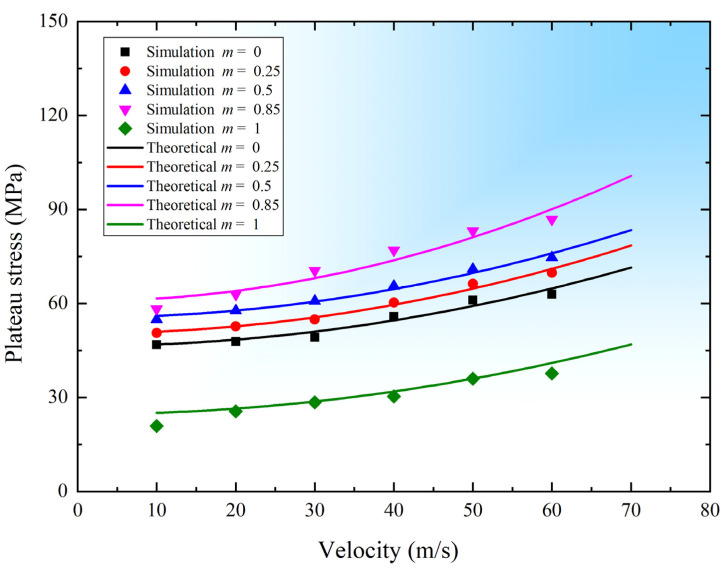
Dynamic plateau stress from numerical simulations and theoretical predictions.

Figure 9 compares the plateau stress from the simulations with that predicted by the theoretical model of Equation (6). It can be noted that the theoretical predictions agree well with the finite element simulation results. It also indicates that the length coefficient m has a significant influence on the dynamic plateau stress, which first increases and then decreases as m increases, with the maximum value occurring at *m* = 0.85. Equations (7)–(11) provides a guide for evaluating the energy absorption capacity of 3D modified FCC lattices under dynamic loading conditions.

### 3.4. Energy Absorption Properties

The energy absorber is designed to achieve several objectives, such as maximizing energy absorption and specific energy absorption, a long and smooth plateau stage, and developing a stable deformation mode. Correspondingly, several indicators have been proposed to evaluate the energy-absorbing properties. Among them, the most commonly used are energy absorption (EA), plateau stress, crushing force efficiency (CFE), and specific energy absorption (SEA).

EA can be obtained by integrating stress σ across the strain ranging from 0 to ε [33,34] (Equation (12)):

(12) $$\mathrm{EA}=\int_{0}^{\varepsilon }\sigma \left(\varepsilon \right)d\varepsilon$$

SEA is another indicator defined by dividing EA with the effective density and is more frequently used for the evaluation of energy absorption capacity since the relative density has a great influence. It can be expressed as Equation (13):

(13) $$\mathrm{SEA}=\frac{\int_{0}^{\varepsilon }\sigma \left(\varepsilon \right)d\varepsilon }{\rho_{R}\rho_{s}}$$

where $\sigma_{R}$ is the relative densities, $\sigma_{s}$ is the constitutive material density.

**Figure 10:**
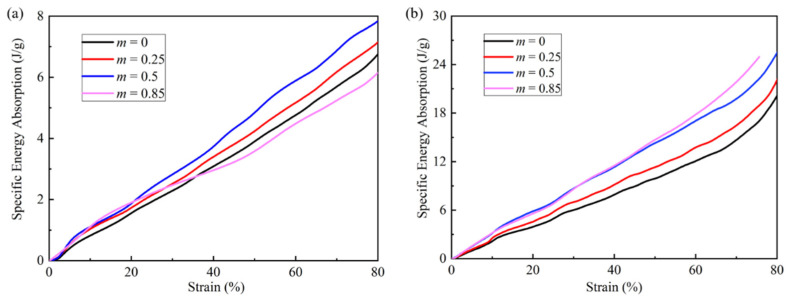
Specific energy absorption of 3D modified FCC lattices with different m under (**a**) quasi-static loading and (**b**) high-speed loading at velocity *V* = 60 m/s.

The SEAs of 3D modified FCC lattices with different m under quasi-static and high-speed loading conditions are compared with each other in Figure 10. It can be seen that SEA strongly depends on the m. The SEA shows a trend of first increasing and then decreasing as m increases under the quasi-static compression, taking the maximum at *m* = 0.5, with a 31.1% increase over the conventional FCC lattice. However, under the high-speed loading condition, the maximum value of SEA takes place at *m* = 0.85, with a 49.3% increase over the conventional FCC lattice. Since the buckling deformation mode can be effectively avoided for the large length coefficient, the energy-absorbing properties of the FCC lattice can be further improved under the high-speed loading condition.

In conclusion, the proposed modified FCC lattice reveals tremendous potential for applications in energy absorption and load-bearing under both quasi-static compression and dynamic impact loading.

## 4. Discussion

In this paper, the mechanical properties and compression behaviors for 2D and 3D modified FCC lattice with different length coefficients under high-speed loading are compared with the results under quasi-static compression. Further, an analytical model for predicting the plateau stresses under different crushing velocities is developed based on the empirical expression in the literature. Some conclusions can be drawn as follows:

1. Under high-speed loading, the global buckling deformation mode of the simple cubic lattice structure and MFCC lattice structure with a larger length coefficient is restrained.
2. The energy absorption and load-bearing capacity of modified FCC lattice is particularly influenced by the length coefficient *m*. At appropriate *m*, the specific energy absorption of MFCC lattice can be improved 49.3% over the conventional FCC lattice under high-speed impact loading.
3. The empirical formula was provided, which can accurately predict the plateau stress for the 3D modified FCC lattice under dynamic loading.

## Data Availability

No applicable.
